# The total-breath method yields higher values of DLCO and TLC than the conventional method

**DOI:** 10.1186/s12890-024-02932-y

**Published:** 2024-03-13

**Authors:** Rudolf A. Jörres, Christian Buess, Andreas Piecyk, Bruce Thompson, Sanja Stanojevic, Helgo Magnussen

**Affiliations:** 1 Institute and Outpatient Clinic for Occupational, Social and Environmental Medicine, University Hospital, LMU Munich, Comprehensive Pneumology Center Munich (CPC-M), German Center for Lung Research (DZL), Ziemssenstraße 5, Munich, 80336 Germany; 2 Ndd Medical Technologies, Zurich, Switzerland; 3 Pneumology Centre Hirslanden, Zurich, Switzerland; 4 Melbourne School of Health Science, The University of Melbourne, Victoria, Australia; 5 Department of Community Health and Epidemiology, Faculty of Medicine, Dalhousie University, Nova Scotia, Canada; 6 Pulmonary Research Institute at LungenClinic Grosshansdorf, Grosshansdorf, Germany

**Keywords:** Diffusing capacity, Lung volume measurements, Pulmonary function testing, Chronic obstructive pulmonary disease

## Abstract

**Background:**

The 2017 ATS/ERS technical standard for measuring the single-breath diffusing capacity (DLCO) proposed the “rapid-gas-analyzer” (RGA) or, equivalently, “total-breath” (TB) method for the determination of total lung capacity (TLC). In this study, we compared DLCO and TLC values estimated using the TB and conventional method, and how estimated TLC using these two methods compared to that determined by body plethysmography.

**Method:**

A total of 95 people with COPD (GOLD grades 1–4) and 23 healthy subjects were studied using the EasyOne Pro (ndd Medical Technologies, Switzerland) and Master Screen Body (Vyaire Medical, Höchberg, Germany).

**Results:**

On average the TB method resulted in higher values of DLCO (mean ± SD Δ = 0.469 ± 0.267; 95%CI: 0.420; 0.517 mmol*min-1*kPa-1) and TLC (Δ = 0.495 ± 0.371; 95%CI: 0.427; 0.562 L) compared with the conventional method. In healthy subjects the ratio between TB and conventional DLCO was close to one. TLC estimated using both methods was lower than that determined by plethysmography. The difference was smaller for the TB method (Δ = 1.064 ± 0.740; 95%CI: 0.929; 1.199 L) compared with the conventional method (Δ = 1.558 ± 0.940; 95%CI: 1.387; 1.739 L). TLC from body plethysmography could be estimated as a function of TB TLC and FEV_1_ Z-Score with an accuracy (normalized root mean square difference) of 9.1%.

**Conclusion:**

The total-breath method yielded higher values of DLCO and TLC than the conventional analysis, especially in subjects with COPD. TLC from the total-breath method can also be used to estimate plethysmographic TLC with better accuracy than the conventional method.

The study is registered under clinicaltrial.gov NCT04531293.

## Results in short

The 2017 ERS/ATS standards for single-breath carbon monoxide uptake in the lung proposed the “total-breath method” for the single-breath maneuver. We found that this method yielded higher values of DLCO and TLC than the conventional method.

## Introduction

**Fig. 1:**
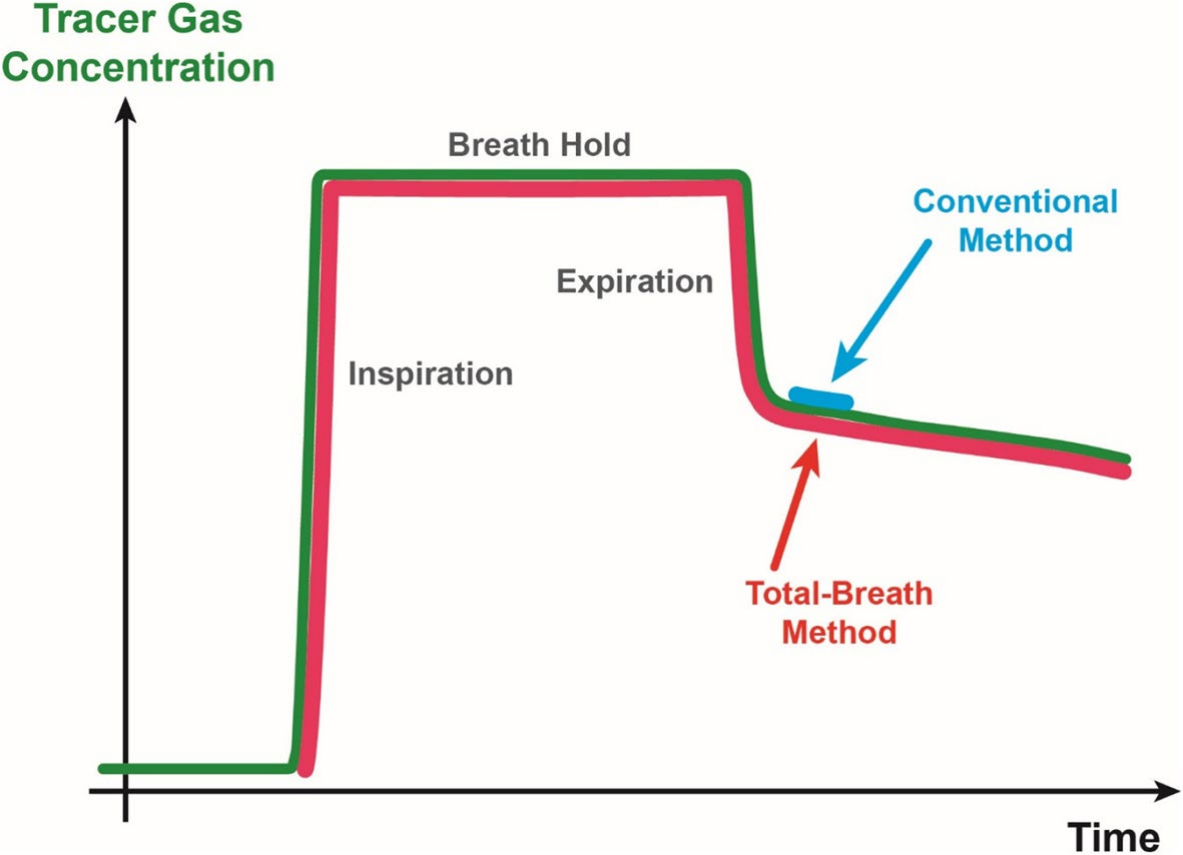
Illustration of the two approaches regarding the total-breath method (TB) using a rapid gas analyzer (RGA) and the conventional sampling (CS) of the inert tracer gas during expiration in the single-breath assessment of CO diffusing capacity of the lung. Although the RGA provides a continuous signal over the whole course of expiration, in the CS method only a defined sample of 500 mL is used (see Materials and methods)

The single-breath diffusing capacity (DLCO) of the lung for carbon monoxide is a well-established functional measure with multiple clinical applications [1, 2]. In 2017, the ATS/ERS published recommendations for its measurement covering technical and methodological requirements. In addition to the conventional method, the technical standard described a procedure for the determination of alveolar volume (VA) in the assessment of diffusing capacity that was introduced in 1985 [3] and then [4] named the rapid gas analyzer (RGA) method, or alternatively [5] total-breath (TB) method. Instead of considering only an average value from a limited sample volume as in conventional sampling (CS), the total-breath method relies on measuring the entire course of exhaled tracer gas concentration, using a rapid gas analyzer (RGA). VA and the corresponding total lung capacity (TLC) are then derived from a mass-balance equation using the entire concentration curve (Fig. 1) [4]. As the total-breath method uses more of the available measured data, it offers the prospect to provide more accurate estimates of lung volume than the conventional method. Since the value of DLCO depends on that of VA, a change in method would also change DLCO values.

Since the values of TLC estimated via gas dilution are affected by inhomogeneity of ventilation, a key feature of COPD [6], the observed differences between the total-breath and the conventional method should become greater in people with more severe COPD. Moreover, the well-known difference between TLC measured via gas dilution and TLC measured via body plethysmography [7] might become smaller with the total-breath method, potentially improving the estimation of plethysmographic TLC from gas dilution.

We aimed to determine the impact of using the total-breath method in healthy subjects and those with COPD of different severities, by comparing DLCO and TLC estimated using the conventional and total-breath method. TLC values were also compared with those measured by body plethysmography.

## Materials and methods

### Recruitment of the study population

Consecutive patients with the clinical diagnosis of COPD [8] were recruited from the pulmonary department at the Hirslanden Hospital Zurich, Switzerland. Healthy subjects, who had no history of respiratory disease, were recruited from hospital staff. Subjects with other types of lung disease than COPD were excluded. The study protocol (clinicaltrial.gov NCT04531293, first trial registration 28/08/2020) was approved by the local ethics committee (Kantonale Ethikkommission Zürich (Cantonal Ethics Committee), BASEC 2020–02139), and all participants gave their written informed consent. All experiments were performed in accordance with relevant guidelines and regulations, in particular the Declaration of Helsinki.

### Assessments

Spirometry was performed using the EasyOne Pro (ndd Medical Technologies, Switzerland) following international recommendations [9]. Forced expiratory volume in 1 s (FEV_1_), forced vital capacity (FVC) and their ratio FEV_1_/FVC were reported, with predicted values from GLI (Global Lung Function Initiative) [10]. Lung volume from body plethysmography (TLC_body_) was estimated using the Master Screen Body (Vyaire Medical, Höchberg, Germany). Diffusing capacity for carbon monoxide (CO) was determined in a single-breath maneuver using the EasyOne Pro (ndd Medical Technologies, Switzerland) and the integrated rapid gas analyzer. The single-breath maneuver was performed twice, separated by at least 5 min [11], and expiration was terminated if a volume plateau was reached. Data was analyzed conventionally by averaging over a defined sample of expiratory volume (conventional sample of 500 mL, CS), or utilizing the total time course of in- and expiration (total-breath, TB) for helium concentration (see Fig. 1). The conventional analysis was available in the EasyOne Pro device (software version V3.7.2.2), which has been shown to yield reliable values in previous studies [12–15]. The total-breath analysis was performed via a custom algorithm after export of the raw data, using the entire tracer gas (helium) concentration during inspiration and expiration (see Fig. 1) within a mass-balance equation. DLCO and TLC were calculated using the residual concentration of helium in the exhaled air as measured at the start of each test. VA was related to TLC by dead space computed as 2.2 mL/kg body weight for BMI < 30 kg/m^2^, and by height^2^ (in m) divided by 189.4 for BMI ≥ 30 kg/m^2^ [4]. Predicted values were taken from the Global Lung Function Initiative reference equations [10, 16].

### Statistical analysis

Continuous data were summarized as mean values and standard deviations (SD). Bland–Altman plots were used to assess the agreement between estimates of DLCO and TLC obtained by different methods. Pearson’s correlation coefficients and linear regression analysis were employed to quantify associations between a set of explanatory variables (sex, age, height, BMI, Z-Scores of FEV_1_ or FEV_1_/FVC) and the differences between the values of TLC or DLCO determined by the two different methods, whereby FEV_1_ or FEV_1_/FVC were tested separately due to their collinearity. A similar approach was followed with plethysmographic TLC as dependent variable. Adjusted R^2^ values and residual standard deviations (RSD) were used to quantify the accuracy of predictions. Groups were compared using the unpaired t-test, or analysis of variance (ANOVA) with post-hoc comparisons according to Duncan in case of more than two independent groups. Outcome values estimated in the same subject were compared using the paired t-test.

The concordance between variables was quantified by their normalized root mean square difference (NSD), defined as standard deviation of the individual differences between two variables divided by their mean value [17]. This is a measure of the residual error of estimation of one variable from another variable after removing their potential systematic difference (bias). The sample size was based on findings by Horstman et al. [5]. Following the aim to detect a difference of the magnitude of 50% of its standard deviation in three pairwise comparisons at a total alpha of 0.05 and a power of 0.95, at least 69 subjects were needed; due to the probably higher variability particularly in patients with severe COPD we aimed at a final size of *n* = 120. Statistical significance was assumed for a type I error of *p* < 0.05. All statistical analyses were performed using the package SPSS (Version 26, IBM, Armonk, NY, USA).

## Results

### Study population

Data from 23 healthy subjects and 95 patients with COPD were available for analysis (Table 1). Subjects with airflow obstruction included all four GOLD grades; with more subjects with GOLD 1 and 2 compared to 3 and 4 (Table 1).

**Table 1:** Characteristics of the patients

Variable	Group		
	**Healthy**	**GOLD 1/2**	**GOLD 3/4**
n	23	64	31
Age (y)	38.6 ± 12.4	71.9 ± 9.9	72.4 ± 6.8
Sex (m/f) (%)	61% / 39%	58% / 42%	45% / 55%
Height (m)	173.5 ± 11.7	171.1 ± 8.2	166.9 ± 8.7
BMI (kg/m^2^)	23.1 ± 2.4	25.2 ± 4.2	25.4 ± 3.7
FEV_1_ (L)	3.93 ± 0.87	1.82 ± 0.50	0.93 ± 0.24
FVC (L)	5.02 ± 1.09	3.30 ± 0.79	2.41 ± 0.58
FEV_1_ (% predicted, GLI)	102.1 ± 7.4	66.7 ± 11.9	37.7 ± 8.5
FVC (% predicted, GLI)	106.8 ± 11.9	92.8 ± 16.1	74.9 ± 15.8
FEV_1_ (Z-score, GLI)	0.180 ± 0.593	-1.929 ± 0.671	-3.495 ± 0.601
FVC (Z-score, GLI)	0.509 ± 0.823	-0.441 ± 0.966	-1.568 ± 1.035
FEV_1_/FVC (Z-score, GLI)	-0.511 ± 0.817	-2.395 ± 0.988	-3.853 ± 0.705
DLCO_CS_ (mmol*min^-1^*kPa^-1^)	10.47 ± 3.02	5.60 ± 2.15	3.67 ± 1.73
DLCO_TB_ (mmol*min^-1^*kPa^-1^)	10.82 ± 3.03	6.06 ± 2.20	4.23 ± 1.80
VA_CS_ (L)	6.19 ± 1.20	5.26 ± 1.20	4.19 ± 0.82
VA_TB_ (L)	6.41 ± 1.21	5.75 ± 1.05	4.94 ± 1.11
TLC_CS_ (L)	6.34 ± 1.20	5.41 ± 0.97	4.34 ± 0.82
TLC_TB_ (L)	6.57 ± 1.24	5.89 ± 1.06	5.07 ± 1.14
TLC_body_ (L)	7.12 ± 1.30	6.84 ± 1.11	6.75 ± 1.55
TLC_body_ – TLC_CS_ (L)	0.78 ± 0.20	1.43 ± 0.69	2.41 ± 1.08
TLC_body_ – TLC_TB_ (L)	0.55 ± 0.22	0.95 ± 0.53	1.68 ± 0.95

COPD patients of GOLD grades 1–4 were pooled into two groups. Mean values ± SD are given. There were significant differences between groups in the distribution of men and women, as well as age, height and BMI (*p* < 0.05 each, ANOVA). Groups were also significantly different from each other (*p* ≤ 0.001 each, ANOVA) in the parameters involving TLC and DLCO, with significant differences between all pairs of groups (*p* < 0.05, Duncan post hoc comparisons). Groups did not significantly differ in TLC_body_

*BMI* body-mass index, *FEV*_*1*_ forced expiratory volume in 1 s, *FVC* forced vital capacity, *DLCO* lung diffusing capacity for carbon monoxide, *VA* alveolar volume, *TLC* total lung capacity, *GLI* Global Lung Function Initiative. Subscripts: *TB* total-breath, *CS* conventional sample, *body* body plethysmography

### Agreement between the duplicate assessments

The time between the duplicate assessments was at minimum 312 s, with a median (quartiles) of 342 (330; 363) seconds. The repeatability of DLCO from the duplicate measurements was high for both the TB and CS method, mean values (95%CI) of differences between 1^st^ and 2^nd^ assessments being -0.002 (-0.069; 0.065) and -0.008 (-0.070; -0.055) mmol*min^-1^*kPa^-1^, respectively. Similar repeatability was observed for TLC, mean values (95%CI) of differences being -0.010 (-0.049; 0.029) and -0.018 (-0.050, 0.014) L for the TB and the CS method, respectively. Therefore, the mean values of the duplicate assessments were used in all subsequent analyses.

### Lung diffusing capacity for CO

**Fig. 2:**
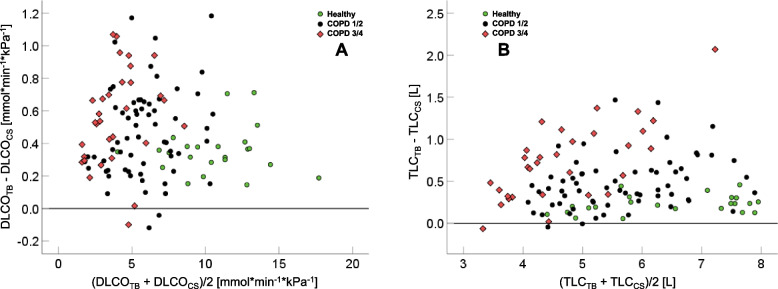
Bland–Altman plots of the difference between DLCO_TB_ and DLCO_CS_ (Panel **A**) or TLC_TB_ and TLC_CS_ (Panel **B**) versus their respective mean values. There was no statistical relationship between the difference and the mean. The lines indicate a difference of zero, and the three groups of healthy control subjects and patients of GOLD grades 1/2 or 3/4 are indicated by different symbols and colors

The correlation between the values of DLCO_TB_ and DLCO_CS_ was high (*r* = 0.994; *p* < 0.001). To elucidate their relationship in detail, Fig. 2A shows a Bland–Altman plot of the difference of DLCO_TB_ and DLCO_CS_ versus their mean value. On average (± SD), DLCO_TB_ was higher by 0.469 ± 0.267 (95%CI: 0.420; 0.517; *p* < 0.001) mmol*min^-1^*kPa^-1^ than DLCO_CS_. The difference was systematically offset and not a function of the magnitude of DLCO, as it was not correlated with the mean value. The NSD between both values was 4.3%.

**Fig. 3:**
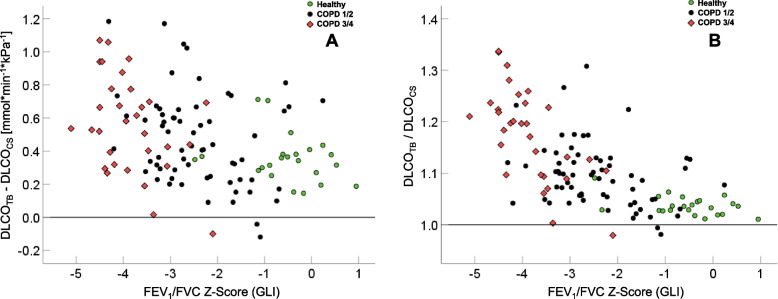
Relationship of the difference between DLCO_TB_ and DLCO_CS_ (panel **A**) and their ratio (panel **B**) to the Z-Score of FEV_1_/FVC. The horizontal lines indicate a ratio of one or a difference of zero, respectively, and the three groups of healthy control subjects and patients of GOLD grades 1/2 or 3/4 are indicated by different symbols and colors

Accounting for sex, age, height and BMI as covariates, the difference between DLCO_TB_ and DLCO_CS_ increased as the FEV_1_/FVC Z-Score decreased (adjusted R^2^ = 0.216, Fig. 3A, *p* < 0.001). A similar result was observed for the ratio of DLCO_TB_ to DLCO_CS_ (adjusted R^2^ = 0.423, Fig. 3B). Both associations were weaker in terms of adjusted R^2^, when using the Z-Score of FEV_1_, indicating the adequacy of FEV_1_/FVC as associated index of airway obstruction.

### Total lung capacity obtained via diffusing capacity manoeuvre

Over the whole range of values, the correlation between TLC_TB_ and TLC_CS_ was high (*r* = 0.953; *p* < 0.001). Figure 2B shows the results of Bland–Altman plots of the difference of TLC_TB_ and TLC_CS_ versus their mean value. Mean (± SD) TLC_TB_ was higher by 0.495 ± 0.371 L than TLC_CS_ (95%CI: 0.427; 0.562 L; *p* < 0.001). Their difference did not significantly correlate with their mean value, and the NSD was 6.7%.

Using sex, age, height and BMI as covariates, again the Z-Score of FEV_1_/FVC was found to be significantly associated with the difference between TLC_TB_ and TLC_CS_ (*p* < 0.001, adjusted R^2^ 0.417) or their ratio (*p* < 0.001, adjusted R^2^ 0.429) and being superior to the Z-Score of FEV_1_.

### Relationship of TLC from single-breath test to TLC from body plethysmography

Both TLC_CS_ and TLC_TB_ were correlated with TLC_body_ (*r* = 0.709 and 0.824, respectively; *p* < 0.001 each). The mean (± SD) difference (95%CI) between TLC_body_ and TLC_CS_ was 1.558 ± 0.940 (95%CI: 1.387; 1.739) L and between TLC_body_ and TLC_TB_ 1.064 ± 0.740 (95%CI: 0.929; 1.199) L; for the differences in single groups see Table 1. The corresponding NSD values were 15.4% for TLC_CS_ and 11.7% for TLC_TB_. Thus, TLC_TB_ was closer to TLC_body_ than TLC_CS_.

**Fig. 4:**
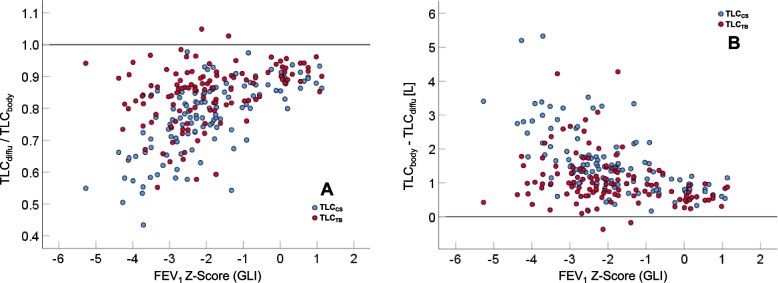
Association between the FEV_1_ Z-Score, and either the ratio (panel **A**) or the difference (panel **B**) between TLC_CS_ determined by the conventional method and TLC_TB_ determined by the total-breath method, divided by TLC_body_ from body plethysmography. The two methods are indicated by different symbols (see insert) and collectively indicated by TLC_diffu_ on the vertical axes. The horizontal line indicates the value of one (panel **A**) or zero (panel **B**)

We then elucidated the relationship between TLC values from gas dilution and plethysmography using regression analysis, assuming that their relationship depended on airway obstruction [18] choosing the Z-Scores of FEV_1_ or FEV_1_/FVC that should always be available in practice, in addition to sex, age, height, BMI as covariates. The dependent variables were the ratios or differences of TLC_CS_ and TLC_TB_ versus TLC_body_. In terms of adjusted R^2^, the Z-Score of FEV_1_ turned out to be slightly superior to that of FEV_1_/FVC for all of these outcomes. Figure 4A shows the ratios TLC_CS_/TLC_body_ and TLC_TB_/TLC_body_ plotted against the Z-Score of FEV_1_, and Fig. 4B the respective differences.

Using linear regression analysis with TLC_TB_ as the only predictor, TLC_body_ could be estimated with a residual error of 0.720 L, which was reduced to 0.621 L by inclusion of the Z-Score of FEV_1_ (adjusted R^2^ 0.676 and 0.756, respectively). Using TLC_CS_ as predictor, the residual errors were 0.895 and 0.745 L, respectively (adjusted R^2^ 0.499, 0.651). The NSD for the estimation of TLC_body_ from TLC_TB_ and FEV_1_ Z-Score was 9.1%, for the estimation from TLC_CS_ and FEV_1_ Z-Score 10.8%.

## Discussion

The recommended total-breath method for estimation of CO diffusing capacity (DLCO) and total lung capacity (TLC) resulted in higher values compared to the conventional analysis. The difference between estimates increased with the degree of spirometrically assessed airway obstruction in patients with COPD. Notably, total-breath TLC more closely agreed with TLC from body plethysmography; the latter could be estimated within less than 10% deviation, when taking into account the degree of airway obstruction.

In clinical practice, the single-breath manoeuvre is primarily performed to determine DLCO, while the assessment of VA, or equivalently TLC, is often secondary. The higher values of DLCO estimated using the TB method are relevant for at least two reasons. First, DLCO is a marker of parenchymal and/or pulmonary vascular dysfunction and airspace destruction, which typically occur in COPD and other lung diseases [1]. In contrast to computed tomography, DLCO has the advantage that it can be assessed repeatedly without radiation and is more affordable. However, in longitudinal analyses a change of method will require careful interpretation regarding comparability of values. Second, higher DLCO values using the TB method may require adaption of categorizations of clinical severity. As the ratio of DLCO_CS_ to DLCO_TB_ was close to one in healthy subjects (Fig. 3A, Table 1), mean predicted values may be kept but quantification of disease severity in COPD in terms of Z-Scores would be affected, if there should be differences in variance and consequently Z-Scores between the two methods; in contrast, grading in percent predicted might be less affected. In addition, further research is needed to reveal whether DLCO_TB_ is superior to DLCO_CS_ in showing better correlations with diagnostic features such as CT-based emphysema scores. No safety- or performance-related issues were observed with the TB method, as the changes solely referred to the mode of data evaluation.

Using the total-breath method, we found higher values for TLC, or equivalently VA, and DLCO. This raises the question to which extent the ratio of DLCO to VA, i.e. the transfer coefficient KCO, was affected. Its value, however, did not change, as the total-breath method altered VA and thereby DLCO by the same factor. The fact that KCO was not affected is relevant, as it is considered as valuable for the identification of causes underlying limitations of gas exchange capacity, in combination with DLCO [1, 2].

For the estimation of TLC via diffusion, the total volume was determined from helium dilution, taking into account the deadspace volume of the apparatus. In clinical practice, most often VA is chosen as volume parameter, requiring the assessment of anatomical deadspace. Moreover, the computation of DLCO involves VA and not TLC. Instead of a fixed deadspace volume of 150 mL, we used volumes computed from either body weight or height, depending on BMI, as previously recommended [4]. An additional analysis using a fixed value of 150 mL showed that, on average, the results regarding DLCO and VA were virtually unaffected by the choice of the method (data not shown). For optimal estimation of dilution, we used the concentration of helium measured in the exhaled air at the start of each test and separated all duplicate assessments by more than 5 min, in line with recommendations [4]. The validity of assessments was underscored by the fact that the duplicate values showed only very small and non-significant differences.

Compared with TLC from body plethysmography, TLC_TB_ differed less than TLC obtained by the conventional method, especially in severe COPD (see Figs. 3 and 4). The difference between TB and plethysmographic TLC could be described as a function of the FEV_1_ Z-Score. The NSD values compared favourably with those reported for different methods providing estimates of plethysmographic TLC [17], including helium and nitrogen washout, CT, and a newly proposed plethysmographic method [17] that showed an NSD of 8–9% compared to standard body plethysmography. Taking NSD as a measure, our observations suggest that the TB method provides more accurate estimates of TLC than the conventional approach, especially in COPD, and that the accuracy is sufficient for clinical purposes.

Regarding plethysmographic TLC, we identified FEV_1_ as best parameter to describe the differences from TLC values obtained by the single-breath method. In a previous study [18], the method of multiple-breath helium dilution was used for comparison with VA assessed via single-breath dilution, and a computational method was described to derive estimates of multiple-breath TLC from single-breath VA. The difference between these values depended on airway obstruction quantified via the ratio FEV_1_/FVC, thereby underlining that spirometric indices can account for the differences between TLC estimates from different procedures. It remains to be assessed whether the ability of TB analysis to provide better estimates also has the potential to improve clinical interpretation and diagnostic insight from functional data.

The difference between plethysmographic and diffusion-based TLC approached a constant offset in healthy subjects with high FEV_1_ Z-scores (see Fig. 4B), which was in the range of 0.5 to 1.0 L. One might assume a homogeneous distribution of the tracer gas over the lungs in healthy subjects after deep inspiration, and thus only a small difference between plethysmographic and diffusion-based volumes, despite sampling errors [19]. As body plethysmographs are not subjected to the same strict requirements of comparability as commercial spirometers, the difference observed could also be due to a bias in the assessment of static lung volumes. For example, when comparing a plethysmograph of the brand that we used with that of another manufacturer, we previously found systematic differences of about 0.67 L in TLC in patients with COPD [20]. It is intriguing that in the study by O’Donnell and co-workers [21] body plethysmographic TLC appeared to be shifted upwards by about 0.5 to 1 L compared to CT-based values of TLC. Thus, potential differences between devices from different manufacturers have to be taken into account when comparing plethysmographic with diffusion-based TLC. As systematic differences between devices by different manufacturers might affect the interpretation of data in terms of predicted values, this topic seem worth of further examination.

### Limitations

The number of patients of GOLD grades 3 and 4 was smaller than that of grades 1 and 2. A higher number of grade 3 and 4 patients might have allowed a more detailed investigation of the factors that explain the difference between plethysmographic TLC and TLC assessed by the TB method, as well as the difference between DLCO values determined by conventional and TB method. On the other hand, this question would probably have benefitted more from additional information such as CT scans in these patients, which were not possible in this study. Such information might be suitable to explain part of the differences between the methods compared in this work. In addition, two of the healthy control subjects showed a Z-Score of less than -1.645 for FEV_1_/FVC. This was a percentage expected by chance, and we left these subjects in the group as we aimed to achieve a continuum of airway obstruction; their omission did not qualitatively change the results. We also considered the fact that the control group was much younger than the COPD group, as an advantage instead of a limitation, since we aimed to include a group for the comparison of the methods, in which optimal lung ventilation and thus minimal differences between parameters could be assumed. From a clinical point of view, the inclusion of patients with restrictive lung disorders or with asthma would have been of interest. Although these patients probably would show similar results as the healthy control subjects, unexpected findings might occur that could be informative for phenotyping. We do not know, whether the TB method is available in other devices than that used in the present study; if that is the case, such devices should be compared to warrant a standard. Moreover, the use of methane instead of helium as tracer gas might result in lower values of TLC particularly in patients with severe COPD and therefore increase the discrepancy from plethysmographic TLC. In addition, it might be helpful to assess TLC_body_ using equipment from different manufacturers, as there is no obligatory standardization regarding these devices.

## Conclusion

The total-breath method for computing TLC and DLCO from a single-breath manoeuvre, as recommended by the 2017 ATS/ERS DLCO technical standard, yielded higher values of DLCO than the conventional analysis, particularly in subjects with COPD. Moreover, TLC values were higher and closer to those from body plethysmography. These could be predicted with an accuracy of less than 10%, especially after inclusion of FEV_1_ as additional variable. The observations suggest that the total-breath method yields more accurate values than the conventional method. Whether it additionally has the potential to improve clinical interpretation and provide novel diagnostic insight from functional data, remains to be studied.

## Data Availability

The authors can be contacted by qualified researchers to get access to the data presented in this work; for this purpose, either the first or the last author can be contacted.

## Abbreviations

ATS: American Thoracic Society
CO: Carbon monoxide
COPD: Chronic obstructive pulmonary disease
CS: Conventional sample
Δ: Delta
DLCO: Transfer factor for CO
ERS: European Respiratory Society
FEV_1_: Forced expiratory volume in 1 s
FVC: Forced vital capacity
GLI: Global Lung Function Initiative
GOLD: Global Initiative for Chronic Obstructive Lung Disease
KCO: Transfer coefficient for CO
NSD: Normalized root mean square difference
RGA: Rapid gas analyzer
SD: Standard deviation
TB: Total-breath
TLC: Total lung capacity
VA: Alveolar volume
